# Comparison of univariate and bivariate Poisson regression methods in the analysis of determinants of female schooling and fertility in Malawi

**DOI:** 10.1186/s12889-024-19816-9

**Published:** 2024-08-22

**Authors:** Eneles Mponda, Tsirizani Mwalimu Kaombe

**Affiliations:** Department of Mathematical Sciences, School of Natural and Applied Sciences, University of Malawi, Zomba, Malawi

**Keywords:** Fertility, Female education, Determinants, Bivariate Poisson regression, Univariate Poisson regression

## Abstract

Recent research has established existence of a correlation between women’s education and fertility, suggesting that they share similar risk factors. However, in many studies, the two variables were analysed separately, which could bias the conclusions by undermining the apparent correlations of such paired outcomes. In this article, the univariate and bivariate Poisson regression models were applied to nationally representative sample of 24,562 women from the 2015-16 Malawi demographic and health survey to examine the risk factors of women’s education levels and fertility. The R software version 4.1.2 was used for the analyses. The results showed that estimates from the bivariate Poisson model were consistent with those obtained from the separate univariate Poisson models. The sizes of estimates of coefficients, their standard errors, *p*-values, and directions were comparable in both bivariate and univariate Poisson models. Using either the univariate or bivariate Poisson model, it was found that the age of a woman at first sexual experience, her current age, household wealth index, and contraceptive usage were significantly associated with both the woman’s schooling and fertility. The study further revealed that ethnicity, religion, and region of residence impacted education level only and not fertility. Similarly, marital status and occupation impacted fertility only and not education. The study also found that higher education levels were linked to a lower number of children, with a strong negative correlation of -0.62 between the two variables. The study recommends using bivariate Poisson regression for analysing paired count response data, when there is an apparent covariance between the outcome variables. The results suggest that efforts by policymakers to achieve the desired women’s sexual and reproductive health in sub-Saharan Africa should be intertwined with improving women’s and girls’ education attainment in the region.

## Introduction

The fertility rate of a woman is defined as the total number of children born alive to her over the course of her lifetime [1]. Over the past years, the world’s total fertility rate has been declining greatly, with an average of 2.3 children per woman from 5 reported in the past across the various regions of the world [2–4]. However, this is not the case in Sub-Saharan Africa (SSA) nations, including Malawi, as the average rate for the region is still high, around 4.6 by 2021 [5]. This may be due to a variety of factors, including cultural and social norms that support high fertility, limited knowledge and lack of access to family planning services, low education attainment, and economic and political stability [6–10]. The term woman schooling generally refers to the formal instruction she receives under the direction of specifically trained teachers [11]. This can be recorded in terms of the total number of years of she attends the formal education during her lifetime [12]. Recently, the average years of women schooling has increased world-wide, from 2.99 in 1970s to 7.02 by 2010 [13]. Some of the factors behind this include delayed marriages, improved household wealth, improved cultural norms, and parental education attainment [12–16]. The determinants of fertility rate vary greatly across cultures and geographical regions [17]. These include education, ethnicity, religion, place of residence, age at first birth, age at first marriage, wealth index, contraceptive usage, post-partum fecundity, and abortion [9, 18–21]. Therefore, high accuracy is required when analysing the fertility and women education data in a particular location.

Previous studies found out that women’s education and fertility are negatively correlated, implying that as the number of years of schooling increases, the number of of children born by a woman tends to decrease, and vice versa [1, 6, 7, 22, 23]. This is the case because people with higher education dedicate more time to learning and skill development, delaying parenthood until they have a stable professional foundation [17]. Researchers often use univariate Poisson regression to separately analyse the determinants of each of the two variables, as they are count variables [19]. Alternatively, other researchers categorise these outcome variables into two or three levels, and apply univariate logistic regression models [7, 10]. However, very little attention has been given to joint modelling of these two count variables. This has potential of overlooking the correlation between the two and bias the estimates. This study therefore applies both univariate and bivariate Poisson regression model to analyse determinants of female schooling and fertility in Malawi using women data obtained from the 2025-16 Malawi Demographic and Health Survey (MDHS). The bivariate Poisson model can simultaneously estimate the correlation between two paired variables and the regression coefficients, without needing to fit the model for each of the two dependent variables being modelled [17, 24, 25]. This not only saves the analyst time but also has a high scientific turnover for estimation. The estimates from the univariate and bivariate regression methods were compared in terms of identifying significant factors using the *p*-values and accuracy based on the sizes of standard errors. Efforts to improve maternal education and fertility outcomes in sub-Saharan Africa through relevant policies need to be augmented by appropriate statistical tools for generating evidence from the avalable data, hence the relevance of this research.

This section is followed by the presentation of methods in Methods section. Thereafter, the results are presented in Results section, followed by the discussion and conclusion in Discussion and Conclusion sections, respectively.

## Methods

### Data

The study used secondary data for women aged 15 to 49 years, which were provided in the “individual records” file of the 2015-16 Malawi Demographic and Health Survey (MDHS). The survey used two-stage stratified sampling, where 850 enumeration areas (clusters) were randomly sampled from across the country at first stage and 27,516 households were sampled from these clusters at second stage using rural and urban stratification [26]. A nationally representative sample of 24,562 women was used in this study. In this present study, interest was on analysing the determinants of female schooling and fertility in Malawi upon fitting univariate and bivariate Poisson models. Therefore, the outcome variables for this study were the total children ever born by a woman and education of the woman in single years.

The analyses used independent variables that were found significant and useful for predicting number of children ever born by a mother and years of schooling in the previous studies [17, 27] and these included: age of a woman at first sex, woman’s current age, region, place of residence (urban or rural), religion, ethnicity, wealth index, contraceptive usage, marital status, and occupation. The analyses were carried out in R version 4.1.2 and data cleaning was performed using STATA version 17. The 2015-16 MDHS dataset is freely available for the users and can be accessed using the website https://dhsprogram.com/data/available-datasets.cfm.

### Univariate and bivariate Poisson models and estimation

Let $Y_{1}$ be the duration of schooling in years and $Y_{2}$ the fertility rate of a woman in the population, with respective measurements, $y_{i1}$ and $y_{i2}$, where $i = 1, 2, ..., n$ is sample point. Further, let $E(Y_{1})=Var(Y_{1})=\lambda _1$ be the mean and variance of $Y_{1}$, and $E(Y_{2})=Var(Y_{2})=\lambda _2$ the mean and variance of $Y_{2}$. If the values of $Y_{1}$ or $Y_{2}$ are independently observed and that $cov(Y_{1},Y_{2})=0$ (i,e., when $Y_{1}$ is not correlated with $Y_{2}$), then each of $Y_{1}$ and $Y_{2}$ has a univariate Poisson distribution with respective parameter $\lambda _{1}$ and $\lambda _{2}$. The two distributions have the following respective mass functions:

1 $$\begin{aligned} f(y_{i1}|\lambda _{1}) & =\frac{e^{-\lambda _{1}}\lambda _{1}^{y_{i1}}}{y_{i1}!}\nonumber \\ & =exp\left[ y_{i1}log \lambda _{1}-\lambda _{1}-log(y_{i1}!)\right] \end{aligned}$$

and

2 $$\begin{aligned} f(y_{i2}|\lambda _{2}) & =\frac{e^{-\lambda _{2}}\lambda _{2}^{y_{i2}}}{y_{i2}!}\nonumber \\ & =exp\left[ y_{i2}log \lambda _{2}-\lambda _{2}-log(y_{i2}!)\right] , \end{aligned}$$

where $y_{i1}, y_{i2}, \lambda _{1}, \lambda _{2} \ge 0$. Therefore, the univariate Poisson distributions in Eqs. (1) and (2) are in standard forms with respective natural parameters $log\lambda _1$ and $log\lambda _2$.

Now, when $Y_{1}$ and $Y_{2}$ are correlated, with $cov(Y_{1},Y_{2})=\lambda _{3}$, where $\lambda _{3} \ne 0$, the random variables $Y_{1}$ and $Y_{2}$ can be assumed to follow a bivariate Poisson distribution with parameters $\lambda _1, \lambda _2, \lambda _3$ [17, 24, 25, 28]. The provabiblity mass function for the bivariate Poisson distribution is given by:

3 $$\begin{aligned} f(y_{i1},y_{i2}|\lambda _{1},\lambda _{2},\lambda _{3}) & = exp(-\lambda _{1}-\lambda _{2}-\lambda _{3})\frac{\lambda _{1}^{y_{i1}}}{y_{i1}!} \frac{\lambda _{2}^{y_{i2}}}{y_{i2}!}\sum \limits _{k=0}^{min(y_{i1},y_{i2})}k!\left( \frac{\lambda _{3}}{\lambda _{1}\lambda _{2}}\right) ^{k}\left( {\begin{array}{c}y_{i1}\\ k\end{array}}\right) \left( {\begin{array}{c}y_{i2}\\ k\end{array}}\right) \nonumber \\ & = exp\left[ y_{i1}log \lambda _{1}+y_{i2}log \lambda _{2}-\lambda _{1}-\lambda _{2}-\lambda _{3}+log\left( \sum \limits _{k=0}^{min(y_{i1},y_{i2})}\frac{k!}{y_{i1}!y_{i2}!}\left( \frac{\lambda _{3}}{\lambda _{1}\lambda _{2}}\right) ^{k}\left( {\begin{array}{c}y_{i1}\\ k\end{array}}\right) \left( {\begin{array}{c}y_{i2}\\ k\end{array}}\right) \right) \right] . \end{aligned}$$

The bivariate Poisson distribution in Eq. (3) is also in standard form with two joint natural parameters $log\lambda _1$ and $log\lambda _2$. When the investigator is interested in estimating the parameters $\lambda _1$ and $\lambda _2$ of the Poisson distribution in either univariate and bivariate case using proxy variables $X_{ij}=(1,x_{i1},x_{i2},\ldots , x_{ip})$ called explanatory variables, then the natural parameters $log\lambda _1$ and $log\lambda _2$ are used to construct a generalised linear relationship with the covariates $X_{j}$ [17, 29].

Therefore, we defined the two univariate Poisson regression models and a bivariate Poisson model as follows:

4 $$\begin{aligned} Y_{i1} =\lambda _{i1}({\textbf {x}})+\epsilon _{i1}, \end{aligned}$$

5 $$\begin{aligned} Y_{i2} =\lambda _{i2}({\textbf {x}})+\epsilon _{i2}, \end{aligned}$$

and

6 $$\begin{aligned} Y_{iw} & =\lambda _{iw}({\textbf {x}})+\epsilon _{iw}; \qquad w = 1, 2, \nonumber \\ \lambda _{i3} & =q({\textbf {x}}). \end{aligned}$$

The term $Y_{iw}\ (w = 1, 2)$ is the univariate count response for the case of univariate model in Eq. (4) or (5) and it is the marginal count outcome in the birvariate Poisson model in Eq. (6). Similarly, $\lambda _{iw}({\textbf {x}})\ (w = 1, 2)$ is the conditional expectation of $Y_{i1}$ and $Y_{i2}$ given *X* when viewed from univariate models in Eqs. (4) and (5). While $\lambda _{iw}({\textbf {x}})$ is the marginal conditional expectation of paired counts $(Y_{i1},Y_{i2})$ given covariates for bivariate model in Eq. (6). The quantity $\epsilon _{iw}\ (w = 1, 2)$ is the model’s error term, capturing the variation in the response $Y_{iw}$ that could not be explained by the use of the covariates *X* in either univariate or bivariate model. The additional marginal model $\lambda _{i3}$ in Eq. (6) is the covariance term that measured the dependence between $Y_{i1}$ and $Y_{i2}$ based on the model. Further, we constricted the row vector of explanatory variables as ${\textbf {x}}=(1,x_{i1},x_{i2},\ldots , x_{ip})$ [17, 29].

Assuming that expected value of the model’s error term, $\epsilon _{iw}\ (w = 1, 2)$ is zero in each case, the conditional expected value of $Y_{iw}$ given ${\textbf {x}}$ is just $\lambda _{iw}({\textbf {x}})$. This is the part linking the model with covariate ${\textbf {x}}$ [17, 29]. We can thus present this simplified form of the three models as follows:

7 $$\begin{aligned} \lambda _{i1}({\textbf {x}})=exp({\textbf {x}}\beta ), \end{aligned}$$

8 $$\begin{aligned} \lambda _{i2}({\textbf {x}})=exp({\textbf {x}}\beta ), \end{aligned}$$

and

9 $$\begin{aligned} \lambda _{i1}({\textbf {x}}) & =exp({\textbf {x}}_1\beta ),\nonumber \\ \lambda _{i2}({\textbf {x}}) & =exp({\textbf {x}}_2\beta )\nonumber \\ \lambda _{i3} & =q({\textbf {x}}), \end{aligned}$$

where $\beta =(\beta _0,\beta _1,\ldots ,\beta _p)$ is the column vector of model parameters, and ${\textbf {x}}_r=(1,x_{i1},x_{i2},\ldots ,x_{ip})\ (r = 1, 2)$ is the row vector of covariates observed for the *i*-th woman that are used in the respective marginal model. Taking the logarithm of each conditional expectation given in models in Eqs. (7-9) gives the link functions derived probability distribution functions presented in Eqs. (1-3) and these provide direct linear relationship with the covariates ${\textbf {x}}$ being used to estimate the count outcome variable $Y_{iw}$ [17].

The forms in Eqs. (7-9) of the univariate and bivariate Poisson regression models as well as the probability mass functions in Eqs. (1-3) were useful for deriving the respective likelihood functions of the the models. The likelihood functions are given by:

10 $$\begin{aligned} L(\beta ) & =\prod _{i=1}^{n}exp\left[ y_{i1}log\lambda _{1}({\textbf {x}})-\lambda _{1}({\textbf {x}})-log(y_{i1}!)\right] \nonumber \\ & =exp\left[ \sum \limits _{i=1}^{n} \left( y_{i1} {\textbf {x}}\beta -exp({\textbf {x}}\beta )-log(y_{i1}!)\right) \right] , \end{aligned}$$

11 $$\begin{aligned} L(\beta ) & =\prod _{i=1}^{n}exp\left[ y_{i2}log\lambda _{2}({\textbf {x}})-\lambda _{2}({\textbf {x}})-log(y_{i2}!)\right] \nonumber \\ & =exp\left[ \sum \limits _{i=1}^{n} \left( y_{i2} {\textbf {x}}\beta -exp({\textbf {x}}\beta )-log(y_{i2}!)\right) \right] , \end{aligned}$$

and

12 $$\begin{aligned} L(\beta ) & =\prod _{i=1}^{n}exp\left[ y_{i1}ln\lambda _{1}({\textbf {x}})+y_{i2}ln\lambda _{2}({\textbf {x}})-\lambda _{1}({\textbf {x}})-\lambda _{2}({\textbf {x}})-q({\textbf {x}})+log \left( \sum \limits _{k=0}^{min(y_{i1},y_{i2})}\frac{\lambda _{1}({\textbf {x}})^{-k}\lambda _{2}({\textbf {x}})^{-k}q({\textbf {x}})^{k}}{k!(y_{i1}-k)!(y_{i2}-k)!}\right) \right] \nonumber \\ & =exp\left[ \sum \limits _{i=1}^{n} \left( y_{i1} {\textbf {x}}_1\beta +y_{i2} {\textbf {x}}_2\beta -exp({\textbf {x}}_1\beta )-exp({\textbf {x}}_2\beta )-q({\textbf {x}})+log \left( \sum \limits _{k=0}^{min(y_{i1},y_{i2})}\frac{exp({\textbf {x}}_1\beta )^{-k}exp({\textbf {x}}_2\beta )^{-k}q({\textbf {x}})^{k}}{k!(y_{i1}-k)!(y_{i2}-k)!}\right) \right) \right] . \end{aligned}$$

The respective log-likelihood functions for the univariate and bivariate models are given by:

13 $$\begin{aligned} l(\beta )=\sum \limits _{i=1}^{n} \left[ y_{i1} {\textbf {x}}\beta -exp({\textbf {x}}\beta )-log(y_{i1}!)\right] , \end{aligned}$$

14 $$\begin{aligned} l(\beta )=\sum \limits _{i=1}^{n} \left[ y_{i2} {\textbf {x}}\beta -exp({\textbf {x}}\beta )-log(y_{i2}!)\right] , \end{aligned}$$

and

15 $$\begin{aligned} l(\beta )=\sum \limits _{i=1}^{n} \left[ y_{i1} {\textbf {x}}_1\beta +y_{i2} {\textbf {x}}_2\beta -exp({\textbf {x}}_1\beta )-exp({\textbf {x}}_2\beta )-q({\textbf {x}})+log \left( \sum \limits _{k=0}^{min(y_{i1},y_{i2})}\frac{exp({\textbf {x}}_1\beta )^{-k}exp({\textbf {x}}_2\beta )^{-k}q({\textbf {x}})^{k}}{k!(y_{i1}-k)!(y_{i2}-k)!}\right) \right] . \end{aligned}$$

By taking the partial derivatives of the log-likelihood functions in Eqs. (13-15) with respect to $\beta$, we arrived at the following respective score vectors:

16 $$\begin{aligned} \frac{\partial {l(\beta )}}{\partial {\beta }}= \sum \limits _{i=1}^{n}\left[ {\textbf {x}}(y_{i1}-exp({\textbf {x}}\beta ))\right] , \end{aligned}$$

17 $$\begin{aligned} \frac{\partial {l(\beta )}}{\partial {\beta }}= \sum \limits _{i=1}^{n}\left[ {\textbf {x}}(y_{i2}-exp({\textbf {x}}\beta ))\right] , \end{aligned}$$

and

18 $$\begin{aligned} \frac{\partial {l(\beta )}}{\partial {\beta _{x1}}} & = \sum \limits _{i=1}^{n}\left[ {\textbf {x}}_1(y_{i1}-exp({\textbf {x}}_1\beta ))-\frac{\sum \nolimits _{k=0}^{min(y_{i1},y_{i2})} \frac{{\textbf {x}}_1(exp({\textbf {x}}_1\beta ))^{-k} (exp({\textbf {x}}_2\beta ))^{-k}(q(x))^k }{(k-1)!(y_{i1}-k)!(y_{i2}-k)!}}{\sum \nolimits _{k=0}^{min(y_{i1},y_{i2})} \frac{(exp({\textbf {x}}_1\beta ))^{-k} (exp({\textbf {x}}_2\beta ))^{-k}(q(x))^k }{k!(y_{i1}-k)!(y_{i2}-k)!}}\right] \nonumber \\ \frac{\partial {l(\beta )}}{\partial {\beta _{x2}}} & = \sum \limits _{i=1}^{n}\left[ {\textbf {x}}_2(y_{i2}-exp({\textbf {x}}_2\beta ))-\frac{\sum \nolimits _{k=0}^{min(y_{i1},y_{i2})} \frac{{\textbf {x}}_2(exp({\textbf {x}}_1\beta ))^{-k} (exp({\textbf {x}}_2\beta ))^{-k}(q(x))^k }{(k-1)!(y_{i1}-k)!(y_{i2}-k)!}}{\sum \nolimits _{k=0}^{min(y_{i1},y_{i2})} \frac{(exp({\textbf {x}}_1\beta ))^{-k} (exp({\textbf {x}}_2\beta ))^{-k}(q(x))^k }{k!(y_{i1}-k)!(y_{i2}-k)!}}\right] . \end{aligned}$$

The subscripts in $\beta _{x1}$ and $\beta _{x2}$ for the score functions in Eq. (18) were meant to emphasize the fact that the differentiation was with repect to parameters in the respective marginal models of the bivariate model [17]. The maximum likelihood estimates $\hat{\beta }$ in the respective model were obtained by solving for $\beta$ when the score vectors were equated to zero. Each of such equations was not in closed form, hence numerical approximations were used. This was done with the help of the R software package VGAMdata supported by the *vglm* function [30]. The value $\hat{\beta }$ represented the effect of a unit increase in the covariate *X* on the logarithm of the expected number of schooling years or children born by a woman adjusting for the other predictor variables in the model. Since the covariance term, $\lambda _{i3}$, in the bivariate Poisson model in Eq. (6) is regarded as a nuisance parameter in the likielihoood function in Eq. (12), it was estimated after the model had been fitted to the data using the estimates of the other parameters [17]. The standardised form of $\lambda _{i3}$, which is the correlation of $Y_{1}$ and $Y_{2}$, was used in this study, and it was estimated through Spearman’s rank correlation method [17].

### Model comparison

The overall goodness of fit of each model to the data was tested using the deviance statistic $D(y_{iw},\hat{\lambda }({\textbf {x}}))=\sum \nolimits _{i=1}^{n}\sum \nolimits _{w=1}^{2} 2[l(\hat{\lambda }_{Fw};y_{iw})- l(\hat{\lambda }_{Rw};y_{iw})]$, where $\hat{\lambda }_{Rw}$ represented the ML estimates in reduced null model (i.e. a model without covariates), while $\hat{\lambda }_{Fw}$ the estimates in full model with covariates, and $l(\hat{\lambda })$ the log-likelihood [29, 31]. The deviance measure has a Chi-square distribution. The null hypothesis in the univariate model case was that the variable $Y_{1}$ or $Y_{2}$ followed univariate Poisson probability distribution. Whereas, in the bivariate model case, the null hypothesis was that the pair $(Y_{1}, Y_{2})$ had a bivariate Poisson distribution. Depending on the estimated deviance statistic and its degrees of freedom, a decision on each hypothesis was made at 5% significance level about the overall fit of each model to the data. Further, the quality of the parameter estimates $\hat{\beta }$ from each model was assessed using the sizes of the standard errors of the estimates. The smaller the standard errors from a particular model the better the model.

## Results

### Data summary

The summary of the 2015-16 MDHS survey data is given in Table 1. The majority of the women completed 1-8 years of education, followed by 9 years and above, and then women who had not attended formal education. On fertility, most of the women had born 1-4 children, followed by 5 children and above, and then no child. The cases of not attending formal education were much concentrated in *Lomwe*, *Yao*, and *Sena* tribes, other religion, poor households, separated, domestic workers, contraceptive users, rural-based, and women from southern region. While education range of 1 to 8 years was dorminant in *Chewa* and *Nyanja* tribes, Muslim, poor households, married, domestic workers, contraceptive users, rural areas, and women from northern region. For the 9 years and avobe schooling bracket, there were mostly *Tumbuka*, *Tonga*, and other tribes, Christian, rich households, unmarried, professional workers, contraceptive non-users, urban-based, and women from northern region.

**Table 1:** Distribution of schooling years and fertility by woman’s socio-demographic features, 2015-16 MDHS

		Years of schooling				Children ever born			
Characteristic	n (%)	0 (%)	1-8 (%)	9+ (%)	$\varvec{\chi }^{\varvec{2}}$ *p*-val	0 (%)	1-4 (%)	5+ (%)	$\varvec{\chi }^{\varvec{2}}$ *p*-val
Overall sample	24,562 (100)	2,988 (12.2)	15,355 (62.5)	6,219(25.3)		5,574 (22.7)	13,162 (53.6)	5,826 (23.7)	
Ethinicity					$<0.0001$				$<0.0001$
Tumbuka/Tonga/other	7,741 (31.5)	527 (6.8)	4,736 (61.2)	2,478 (32.0)		1,771 (22.9)	4,185 (54.1)	1,785 (23.0)	
Lomwe/Yao/Sena	8,388 (34.2)	1,320 (15.7)	5,274 (62.9)	1,794 (21.4)		1,801 (21.5)	4,623 (55.1)	1,964 (23.4)	
Chewa/Nyanja	8,433 (34.3)	1,141 (13.5)	5,345 (63.4)	1,947 (23.1)		2,002 (23.7)	4,354 (51.6)	2,077 (24.7)	
Religion					$<0.0001$				$<0.0001$
None/other	151 (0.6)	58 (38.4)	69 (45.7)	24 (15.9)		23 (15.2)	79 (52.3)	49 (32.5)	
Muslim	2,726 (11.1)	614 (22.5)	1,731 (63.5)	381 (14.0)		530 (19.5)	1,476 (54.1)	720 (26.4)	
Christian	21,685 (88.3)	2,316 (10.7)	13,555 (62.5)	5,814 (26.8)		5,021 (23.2)	11,607 (53.5)	5,057 (23.3)	
Wealth status					$<0.0001$				$<0.0001$
Poor	8,708 (35.5)	1,706 (19.6)	6,338 (72.8)	664 (07.6)		1,491 (17.1)	4,840 (55.6)	2,377 (27.3)	
Middle	4,508 (18.3)	599 (13.3)	3,270 (72.5)	639 (14.2)		928 (20.6)	2,331 (51.7)	1,249 (27.7)	
Rich	11,346 (46.2)	683 (06.0)	5,747 (50.7)	4,916 (43.3)		3,155 (27.8)	5,991 (52.8)	2,200 (19.4)	
Marital status					$<0.0001$				$<0.0001$
Unmarried	5,326 (21.7)	103 (01.9)	3,074 (57.7)	2,149 (40.4)		4,595 (86.3)	710 (13.3)	21 (00.4)	
Married/cohabited	15,952 (64.9)	2,283 (14.3)	10,244 (64.2)	3,425 (21.5)		886 (05.6)	10,336 (64.8)	4,730 (29.6)	
Separated/other	3,284 (13.4)	602 (18.3)	2,037 (62.0)	645 (19.6)		93 (02.8)	2,116 (64.5)	1,075 (32.7)	
Occupation					$<0.0001$				$<0.0001$
Not working	8,422 (32.3)	805 (09.5)	5,261 (62.5)	2,356 (28.0)		3,209 (38.1)	3,906 (46.4)	1,307 (15.5)	
Domestic/Nonformal	14,093 (57.4)	2,095 (14.9)	9,399 (66.7)	2,599 (18.4)		2,059 (14.6)	7,862 (55.8)	4,172 (29.6)	
Professional/formal	2,047 (08.3)	88 (04.3)	695 (34.0)	1,264 (61.7)		306 (14.9)	1,394 (68.1)	347 (17.0)	
Contraceptive use					$<0.0001$				$<0.0001$
Non-user/other	13,574 (55.3)	1,591 (11.7)	8,279 (61.0)	3,704 (27.3)		5,248 (38.7)	5,934 (43.7)	2,392 (17.6)	
User	10,988 (44.7)	1,397 (12.7)	7,076 (64.4)	2,515 (22.9)		326 (03.0)	7,228 (65.8)	3,434 (31.2)	
Place of residence					$<0.0001$				$<0.0001$
Urban	5,247 (21.4)	227 (04.3)	2,215 (42.2)	2,805 (53.5)		1,521 (29.0)	3,082 (58.7)	644 (12.3)	
Rural	19,315 (78.6)	2,761 (14.3)	13,140 (68.0)	3,414 (17.7)		4,053 (21.0)	10,080 (52.2)	5,182 (26.8)	
Region					$<0.0001$				$<0.0001$
Northern	4,803 (19.5)	196 (04.1)	3,015 (62.8)	1,592 (33.1)		1,090 (22.7)	2,598 (54.1)	1,115 (23.2)	
Central	8,417 (34.3)	1,104 (13.1)	5,272 (62.6)	2,041 (24.3)		2,038 (24.2)	4,362 (51.8)	2,017 (24.0)	
Southern	11,342 (46.2)	1,688 (14.9)	7,068 (62.3)	2,586 (22.8)		2,446 (21.6)	6,202 (54.7)	2,694 (23.8)	
Mean age (SD)		35.6 (8.82)	27.4 (9.28)	26.3 (7.57)		18.5 (4.32)	27.6 (6.93)	38.7 (5.74)	
Mean age at 1st sex (SD)		15.8 (2.72)	16.1 (2.61)	17.6 (3.10)		16.7 (3.57)	16.6 (2.60)	15.8 (2.45)	

*SD* Standard deviation, $\chi ^{2}$ *pval* Chi-Square Test *p*-value

Further, no child outcome was more dominant in *Chewa* and *Nyanja* tribes, Christian, rich households, unmarried, non-working, contraceptive non-users, urban areas, and northern region. Whereas the majority of the women who had between 1 to 4 children were from *Lomwe*, *Yao*, and *Sena* tribes, Muslim, poor households, married, professional workers, contraceptive users, urban-based, and women from southern region. Further, having at least 5 children was prevalent in *Chewa* and *Nyanja* tribes, other religion, middle class, separated, domestic workers, contraceptive users, rural areas, and central region. The mean age for sex debut was 16 years, and 2.8 years standard deviation. The Chi-square test results showed that all the included variables were associated with both outcome variables at 5% significance level.

### Univariate and bivariate Poisson models’ estimates

The results in Table 2 are for the maximum likelihood estimates from the univariate and bivariate Poisson models that were fitted to the schooling and fertility data. The deviance statistic results showed that the bivariate Poisson distribution assumption for the two response variables was satisfied by the fitted model. Similarly, the assumption of univariate Poisson distribution for each of the two outcome variables also matched the data well in the separate fitted models. The maximum likelihood findings indicate that the estimates by the sets of the models were similar in size, direction, as well as significance. The models commonly identified household wealth index, contraceptive use, place of residence, age of a woman, and age at sex debut as factors that significantly affect both schooling and fertility. The models also found region, religion, ethnic group as factors that affected female schooling only. While marital status and occupation impacted the fertility only. The standard errors of estimates were similar in both sets of models.

**Table 2:** Effects of women characteristics on schooling and fertility outcomes upon fitting univariate and bivariate Poisson models to 2015-16 MDHS data

	Schooling		Fertility	
Variable	Univariate Poisson Log-Mean (SE, pval)	Bivariate Poisson Log-Mean (SE, pval)	Univariate Poisson Log-Mean (SE, pval)	Bivariate Poisson Log-Mean (SE, pval)
Intercept	1.42 (0.05, $<0.0001$)	1.42 (0.05, $<0.0001$)	-2.17 (0.06, $<0.0001$)	-2.17 (0.06, $<0.0001$)
Ethnicity				
Tumbuka/Tonga/oth^*^				
Lomwe/Yao/Sena	-0.06 (0.01, $<0.0001$)	-0.06 (0.01, $<0.0001$)	-0.01 (0.01, 0.300)	-0.01 (0.01, 0.300)
Chewa/Nyanja	-0.09 (0.01, $<0.0001$)	-0.09 (0.01, $<0.0001$)	0.02 (0.01, 0.083)	0.02 (0.01, 0.083)
Religion				
None/Other^*^				
Muslim	0.15 (0.04, 0.0005)	0.15 (0.04, 0.0005)	0.11 (0.05, 0.016)	0.11 (0.05, 0.016)
Christian	0.35 (0.04, $<0.0001$)	0.35 (0.04, $<0.0001$)	0.02 (0.04, 0.573)	0.02 (0.04, 0.573)
Wealth				
Poor^*^				
Middle	0.18 (0.01, $<0.0001$)	0.18 (0.01, $<0.0001$)	-0.04 (0.01, 0.0002)	-0.04 (0.01, 0.0002)
Rich	0.42 (0.01, $<0.0001$)	0.42 (0.01, $<0.0001$)	-0.11 (0.01, $<0.0001$)	-0.11 (0.01, $<0.0001$)
Marital status				
Unmarried^*^				
Married/cohabited	-0.02 (0.01, 0.012)	-0.02 (0.01, 0.012)	1.98 (0.03, $<0.0001$)	1.98 (0.03, $<0.0001$)
Separated/other	0.004 (0.01, 0.706)	0.004 (0.01, 0.706)	1.90 (0.03, $<0.0001$)	1.90 (0.03, $<0.0001$)
Occupation				
Not working^*^				
Domestic/informal	-0.00 (0.01, 0.956)	-0.00 (0.01, 0.956)	0.04 (0.01, $<0.0001$)	0.04 (0.01, $<0.0001$)
Professional/formal	0.33 (0.01, $<0.0001$)	0.33 (0.01, $<0.0001$)	-0.08 (0.02, $<0.0001$)	-0.08 (0.02, $<0.0001$)
Contraceptive use				
Non-user/other^*^				
User	0.04 (0.01, $<0.0001$)	0.04 (0.01, $<0.0001$)	0.19 (0.01, $<0.0001$)	0.19 (0.01, $<0.0001$)
Place of residence				
Urban^*^				
Rural	-0.18 (0.01, $<0.0001$)	-0.18 (0.01, $<0.0001$)	0.15 (0.01, $<0.0001$)	0.15 (0.01, $<0.0001$)
Region				
Northern^*^				
Central	-0.09 (0.01, $<0.0001$)	-0.09 (0.01, $<0.0001$)	-0.02 (0.01, 0.073)	-0.02 (0.01, 0.073)
Southern	-0.11 (0.01, $<0.0001$)	-0.11 (0.01, $<0.0001$)	-0.02 (0.01, 0.148)	-0.02 (0.01, 0.148)
Current age	-0.02 (0.00, $<0.0001$)	-0.02 (0.00, $<0.0001$)	0.06 (0.00, $<0.0001$)	0.06 (0.00, $<0.0001$)
Age at 1st sex	0.04 (0.00, $<0.0001$)	0.04 (0.00, $<0.0001$)	-0.04 (0.00, $<0.0001$)	-0.04 (0.00, $<0.0001$)
Deviance (DF)	50,512 (24,545)	66,997.24 (49,090)	16,485 (24,545)	66,997.24 (49,090)
Correlation (pval)		-0.62 ($<0.0001$)		

$(*)$ reference category, *pval* *p*-value, *SE* standard error, *DF* degrees of freedom

In particular, it was found that Muslim women had, on average, a 0.15 increase and Christian women had a 0.35 increase in the adjusted logarithm of the mean number of years of schooling compared to those with no religious affiliation. Similarly, women from middle-income households had an average increase of 0.18 in log-mean years of schooling, while women from rich households had an average increase of 0.42 compared to women from poor households. Additionally, professional and formally employed women had an average increase of 0.33 in the duration of schooling compared to non-working women. Moreover, women who used modern contraceptive methods had an average increase of 0.04 in the duration of schooling compared to non-users. An increase in age at first sex corresponded to an average increase of 0.04 in log-mean years of schooling by the woman. However, women from the *Lomwe* or *Yao* or *Sena* and *Chewa* or *Nyanja* tribes had a decrease of 0.06 and 0.09, respectively, in the duration of schooling compared to those from the *Tumbuka* or *Tonga* and other tribes. Women from rural areas experienced an average decrease of 0.18 in the log-mean duration of schooling compared to those in urban locations. Furthermore, women residing in the Central and Southern regions experienced an average decrease of 0.09 and 0.11, respectively, in the log-mean duration of schooling compared to those from the Northern region.

With fertility outcomes, the adjusted log-mean number of children born to Muslim women increased by 0.11 compared to those without religion. Additionally, the log-mean number of children born to married or cohabited and separated women increased by 1.98 and 1.90, respectively, compared to unmarried women. Similarly, the log-mean number of children increased by 0.04 for domestic workers compared to non-working women. Women using modern contraceptive methods had a 0.19 increase in the log-mean number of children born compared to non-users. Women residing in rural areas had a 0.15 increase in the log-mean number of children born compared to those from urban areas. Moreover, a unit increase in a woman’s age corresponded to a 0.06 increase in the log-mean number of children born. Conversely, women from middle and rich households had a 0.04 and 0.11 reduction, respectively, in the log-mean number of children born compared to women from poor households. Additionally, women with professional or formal jobs had a 0.08 reduction in the log-mean number of children born compared to non-working women. Furthermore, a unit increase in age at first intercourse corresponded to a 0.04 decrease in the log-mean number of children born. Without considering the covariates in the model, the log-mean schooling increased by a factor of 1.42, while the log of mean fertility decreased by a factor of 2.17 in the study population.

## Discussion

This study investigated common determinants of fertility and female education duration, the amount of covariance between the two variables, and perfomance of univariate and bivariate Poisson models in the analyses, using the survey data from Malawi. The study found that the two count variables were inversely correlated with a correlation estimate of -0.62. Thus, high values of female education paired with low values of fertility, and vise versa. This is the pattern other studies also observed, and usually attributed it to delayed maternal age that education persuance brings to a woman’s life [17, 21, 23]. The findings showed that the sizes of maximum likelihood estimates, their direction, significance, and standard errors were similar between using univariate and bivariate Poisson regression models on the data. In addition, each model matched its distribution assumption, according to the deviance statistic. The similarity between estimates from the univariate and bivariate Poisson models typically occurs when the equi-dispersion assumption of the model is supported by the data, as observed in other studies [32]. Further, the study observed that age of a woman at first sex, her current age, contraceptive use, household wealth index, and place of residence were significantly associated with both fertility and a woman’s education. The other factors were uncommon, for example, the woman’s marital status and her occupation only impacted her fertiliy and not schooling. While ethnicity, region of stay, and religion only affected the woman’s education level and not fertility. These results align with those found in previous studies conducted in some parts of Africa [1, 13, 20, 21, 33].

Amongst the findings, the current analysis showed that the fertility is higher in rural residents, contraceptive users, domestic workers, Muslims and Christians, married and separated women, and older women. The high risk of fertility in rural areas was also observed in previous studies conducted in sub-Saharan Africa [1, 34]. The limited access to birth control packages coupled with low reproductive health literacy levels could be amongst the factors that promote high fertility in rural areas of the country [20]. The high fertility in contraceptive users is unxpected but popular result in the studied population, and the reasons for this trend are yet to be studied [17]. Most quantitative studies reported clear uncertainties or inconsistences on the role of contraceptive use in preventing unintended pregnancy or child in least developed countries, inviting more research on this subject [35, 36]. Some previous studies suspected that postpartum overlap in cross-sectional surveys might bias the effect of contraceptive use on fertility, which suggest using other alternative designs to generate data for this subject [37].

The association of domestic work and fertility is a proxy one. Most housemaids in the study population are originally from rural locations, who either dropped out of school or divorced [38]. It is these other factors, than domestic work itself, that relate with high fertility as reported in previous studies [10, 17]. More importantly, this study, being based on a cross-sectional survey, did not control for causal inference conditions to establish the causal relationship between domestic work and fertility [39, 40]. The Christians and Muslims form the majority of the population in Malawi, it is therefore not surprising that fertility was higher in this group that non-religious women. It was also not strange for the married women to have higher fertility because they have the increased desire to have children, as observed in previous studies [41]. Similarly, it is natural for older women to have a higher number of children compared to their younger counterparts. Fertility was observed to be low in middle and rich households, and in women with higher age at first sex. This is probably due to high access to modern birth control methods for families that have financial resources, which was observed in other studies [7]. As for age at sex debut, it is observed in previous studies that delaying sex leads to low numbers of children born by the woman, since child bearing is through sex by nature [41].

Furthermore, it was found that the duration of schooling was high in Muslim and Christian women, middle and rich households, professional and formal workers, contraceptive users, and higher age at sex debut. The reasons for wealthier households having higher education are related to those highlighted above, it is to do with access and affordability due to financial resources as reported in previous studies [7]. The high age at first sex provides room for educational attainment as corroborated in many studies, and hence it was not a surprising result [14–16]. The high education observed in professional working class women is a reverse causation, as one needs to be educated first to secure a formal employment. This might also be the case with the positive association observed between contractive use and education. Education attainment improves literacy level and hence knowledge on usage of modern contraceptive methods [16]. The Christian and Muslim women benefit from the parental education attainment in their households as a source of motivation for them to pursue their studies unlike non-religious communities which are also less educated as noted in other studies [12, 15, 16]. The study found that the schooling period was low in *Lomwe*, *Yao*, *Sena*, *Chewa*, and *Njanya* tribes, rural residents, Central and Southern regions of the study location, and older women. Apparently, there is high prevalence of early marriages in the aforementioned cultures as well as regions in Malawi reported in previous research, which might impact the low education attainment observed in these tribes and locations [17, 42]. As stated before, rural residents in sub-Saharan Africa face various socio-economic inequalities like lack of financial resources and unfavourable cultural norms and traditions, that disadvantage them to progress in education [15]. So, it was not strange that this study observed low education levels for women from rural areas in Malawi.

Finally, this study used the bivariate Poisson modelling to consider the relationship between various covariates and a woman’s fertility and her education, while accounting for the correlation between the two dependent variables [17]. However, this model has limitations when it comes to accounting for the clustering of observations for different women within the survey data. Since the data was collected using a cluster design, there is a possibility of dependence among observations of women from the same cluster, which goes against the independence assumption of the maximum likelihood estimation and could introduce bias in estimation [43–45]. To address this issue, it is recommended to utilize generalized linear mixed-effects models for this type of data [46, 47]. For future research, this study suggests applying mixed-effects bivariate Poisson models to these data.

## Conclusion

In this paper, the performance of bivariate and univariate Poisson regression models in finding risk factors of women education and fertility was studied using data from Malawi. The estimates from bivariate Poisson model were consistent with those from separate univariate Poisson models. Both sets of models found the same common factors that were associated with fertility and female schooling, as well as uncommon factors that impacted one of the two variables and not the other. The two variables were highly negatively correlated, with a correlation value of -0.62, thus women with more years of schooling had smaller number of children born and vice versa. The bivariate Poisson model had few advantages over the univariate model. Firstly, apart from maximum likelihood estimates of regression parameters, it could allow estimation of the covariance or correlation between fertility and female education based on the fitted model to data.

Secondly, the bivariate Poisson model was fitted once to the dataset and provided all the estimates that have been reported, which saved time compared to fitting the univariate model twice. On this basis, the study recommends fitting a bivariate Poisson model to paired count data whenever possible. The study provides compelling evidence on the risk factors of female schooling duration and fertility, based on a large, nationally representative sample and relevant statistical methods. However, some findings lacked causal explanations, so the study recommends follow up longitudinal and qualitative study designs to establish causation for variables like domestic work and contraceptive usage, which showed an association with fertility and schooling. Due to the strong correlation between female education and fertility and the various risk factors affecting both variables, the study recommends intertwining policy initiatives aimed at improving women’s sexual and reproductive health in the sub-Saharan African region with appropriate women’s and girls’ education interventions. Future research could apply zero-inflated bivariate Poisson models when analysing similar data due to the presence of zero counts that were observed in the data.

## Data Availability

The 2015-16 MDHS data are publicly and freely available for users at https://dhsprogram.com/data/new-user-registration.cfm.
